# Impact of elastic substrate on the dynamic heterogeneity of WC256 Walker carcinosarcoma cells

**DOI:** 10.1038/s41598-023-35313-2

**Published:** 2023-09-21

**Authors:** Aleksandra Mielnicka, Tomasz Kołodziej, Daniel Dziob, Sławomir Lasota, Jolanta Sroka, Zenon Rajfur

**Affiliations:** 1 Department of Molecular and Interfacial Biophysics, Faculty of Physics, Astronomy and Applied Computer Science, Jagiellonian University, ul. Lojasiewicza 11, 30-348 Kraków, Poland; 2 BRAINCITY, Laboratory of Neurobiology, The Nencki Institute of Experimental Biology, PAS, ul. Ludwika Pasteura 3, 02-093 Warsaw, Poland; 3 Department of Pharmaceutical Biophysics, Faculty of Pharmacy, Jagiellonian University Medical College, ul. Medyczna 9, 30-688 Kraków, Poland; 4 Department of Cell Biology, Faculty of Biochemistry, Biophysics and Biotechnology, Jagiellonian University, ul. Gronostajowa 7, 30-387 Kraków, Poland; 5 Jagiellonian Center of Biomedical Imaging, Jagiellonian University, 30-348 Kraków, Poland

**Keywords:** Cell migration, Tumour heterogeneity, Biological physics

## Abstract

Cellular heterogeneity is a phenomenon in which cell populations are composed of subpopulations that vary in their behavior. Heterogeneity is particularly pronounced in cancer cells and can affect the efficacy of oncological therapies. Previous studies have considered heterogeneity dynamics to be indicative of evolutionary changes within subpopulations; however, these studies do not consider the short-time morphological plasticity of cells. Physical properties of the microenvironment elasticity have also been poorly investigated within the context of cellular heterogeneity, despite its role in determining cellular behavior. This article demonstrates that cellular heterogeneity can be highly dynamic and dependent on the micromechanical properties of the substrate. During observation, migrating Walker carcinosarcoma WC256 cells were observed to belong to different subpopulations, in which their morphologies and migration strategies differed. Furthermore, the application of an elastic substrate (E = 40 kPa) modified three aspects of cellular heterogeneity: the occurrence of subpopulations, the occurrence of transitions between subpopulations, and cellular migration and morphology. These findings provide a new perspective in the analysis of cellular heterogeneity, whereby it may not be a static feature of cancer cell populations, instead varying over time. This helps further the understanding of cancer cell behavior, including their phenotype and migration strategy, which may help to improve cancer therapies by extending their suitability to investigate tumor heterogeneity.

## Introduction

Cellular heterogeneity is a phenomenon defined as the occurrence of distinct cell subpopulations that express different behavior^1^. Differences in cell morphology, protein expression, and other parameters can be widely observed in high-resolution examinations of almost all cells^2^. This diversity between cells is particularly visible in tumors, causing genetic, epigenetic, and environmental heterogeneity^3^. This heterogeneity can significantly impact cancer treatment due to the modified responses of cells^4^ and is related to drug resistance mechanisms^5, 6^. Proper identification of heterogeneity can guide therapeutic decisions^3^ and provides the opportunity to create personalized therapies^4^. The temporal dynamics of cancer cell heterogeneity is a significant factor in tumor evolution and can result from the application of chemotherapeutics^7, 8^. The current literature reports that such dynamics occur across a long time (days to months) that is typically required for cellular mutations. However, the short-term plasticity of heterogeneous cancer cells has not been investigated yet.

Another factor that is not fully understood within the context of cellular heterogeneity is the interaction with the physical microenvironment, which influences pathological and physiological cellular processes. Disruptions in tissue organization can lead to the cancerous transformation of healthy cells caused by modifications in extracellular matrix (ECM)^3^. The interaction between cancer cells and their microenvironment can determine their progression, invasion, metastasis, and migration^9, 10^. The mechanical properties of the microenvironment, particularly its elasticity, as described by Young’s modulus of a substrate and ECM, combined with cellular signals, determine the spatial configuration of cells and provide various biochemical and biophysical stimuli that influence their behavior^11, 12^. Mechanical interactions between cells and the substrate are translated into cytoskeletal-dependent biochemical activities that regulate cellular apoptosis, proliferation, differentiation, and migration^13–19^. These biochemical signals are also reflected in an arrangement of cell’s cytoskeleton which exerts forces on the extracellular environment and modifies cellular phenotypes and morphologies. In certain cases, the phenotypic plasticity of cancer cells can lead to a metastatic transformation that induces cell migration and progression in distant organs^20^. These transformations can result in cell shape rearrangement and the creation of cellular protrusions, which consequently influence cellular migration due to their impacts on the kinematic parameters of cellular motor activity. This indicates that protrusion plays an important role in determining the degree of tumor invasiveness^21^. The wide variety of extracellular signals which determine pathomechanisms and cell transformation^22^ impact cells differently. However, cell heterogeneity makes difficult to link these signals with a particular path of cancerous transformation and progression^18^. Together, these factors highlight the significance of studies investigating the phenotypic and migratory heterogeneity of cancer cells within the context of microenvironment interactions.

The development of biophysical techniques has enabled the observation of extracellular signal effects on the mechanical properties of individual cancer cells, allowing differences to be recorded in relation to non-cancerous cells^18, 23, 24^. The application of elastic polymer substrates made of polyacrylamide (PA) is one method used to distinguish the influence of chemical and physical signals on cellular processes^14, 25^. These substrates can mimic the physical microenvironment created by soft tissues. Using synthetic substrates as biomaterials has led to new perspectives, as they react to dynamic changes in cellular functionality by undergoing deformations due to the forces exerted by cells^14, 26, 27^. PA substrates have already served as a biomimetic substitute for elastic tissues in numerous studies, including ones investigating cancer^28, 29^, the immune system^30, 31^, muscle^32^, and stem cells^33–35^ under various conditions.

The current study investigates the influence of the elastic microenvironment on the heterogeneity of the adherent subline of Walker carcinosarcoma WC256 rat cells. Walker WC256 cells are responsible for malignant tumor progression. Carcinosarcomas primarily metastasize to the bones, causing increased bone resorption and hypercalcemia^36–40^. These cells provide an excellent model for the analysis of metastasis and migration processes due to their phenotypic plasticity and the effectiveness of dynamic two-way transitions between mesenchymal migration and the amoeboid mechanism of cell movement. Previous studies have focused on the comparison of adherent and non-adherent WC256 sublines, discussing their heterogeneity in the context of cell morphology and migration strategy^41, 42^. The current study is focused on the adherent subline, which is composed of cells with different phenotypes identified in previous work. The time-lapse technique used in this study enabled, for the first time, the observation of transitions between different cell subtypes, demonstrating that the heterogeneity of adherent subline of WC256 cells is a dynamic phenomenon. Transitions occurred in a short timeframe (minutes to hours). Additionally, the application of an elastic substrate was found to alter cellular heterogeneity on three levels: the occurrence of different subpopulations, transitions between subpopulations, and the morphology and migration parameters of each subpopulation.

## Results

### Populational heterogeneity of WC256 cells

**Figure 1:**
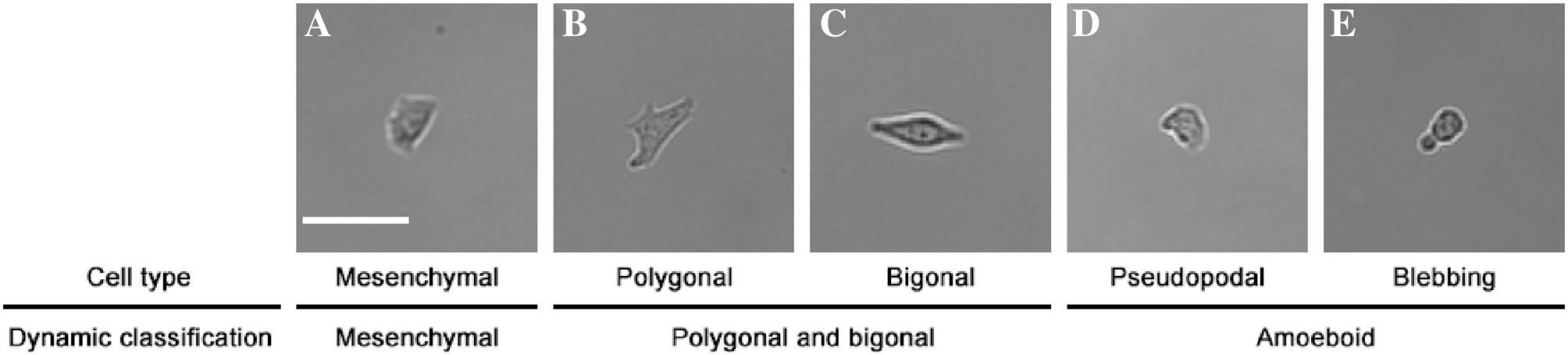
Different subpopulations of WC256 adherent subline. (**A**) Mesenchymal (mesenchymal subpopulation), (**B**) polygonal (polygonal and bigonal subpopulation), (**C**) bigonal (polygonal and bigonal subpopulation), (**D**) pseudopodial (amoeboid subpopulation), (**E**) blebbing (amoeboid subpopulation). Magnification 10x , scalebar = 50 µm. Dynamic behavior of the same cells is presented in Supplementary Video S1.

The morphology and migration strategy of cells were assessed at each time point according to the criteria discussed in the “Materials and methods” section. Figure 1 provides examples of cells from the different subpopulations, described by cell type and dynamics. The migration of these cells is presented in Supplementary Video S1. In addition, preliminary analysis showed that the mesenchymal subpopulation of the WC256 line is comprised of cells that behave differently from each other, even if they meet the criteria to be considered part of the same subpopulation. These cells differed in the presence of a retraction tail, small protrusions, the shape of the leading edge, or migration velocity. Although investigating the diversity of the mesenchymal subpopulation was not the aim of this study, this is a possible avenue for future investigations. Exemplary mesenchymal cells migrating on glass substrates are shown in Supplementary Video S2.

### Dynamic heterogeneity of WC256 cells

**Figure 2:**
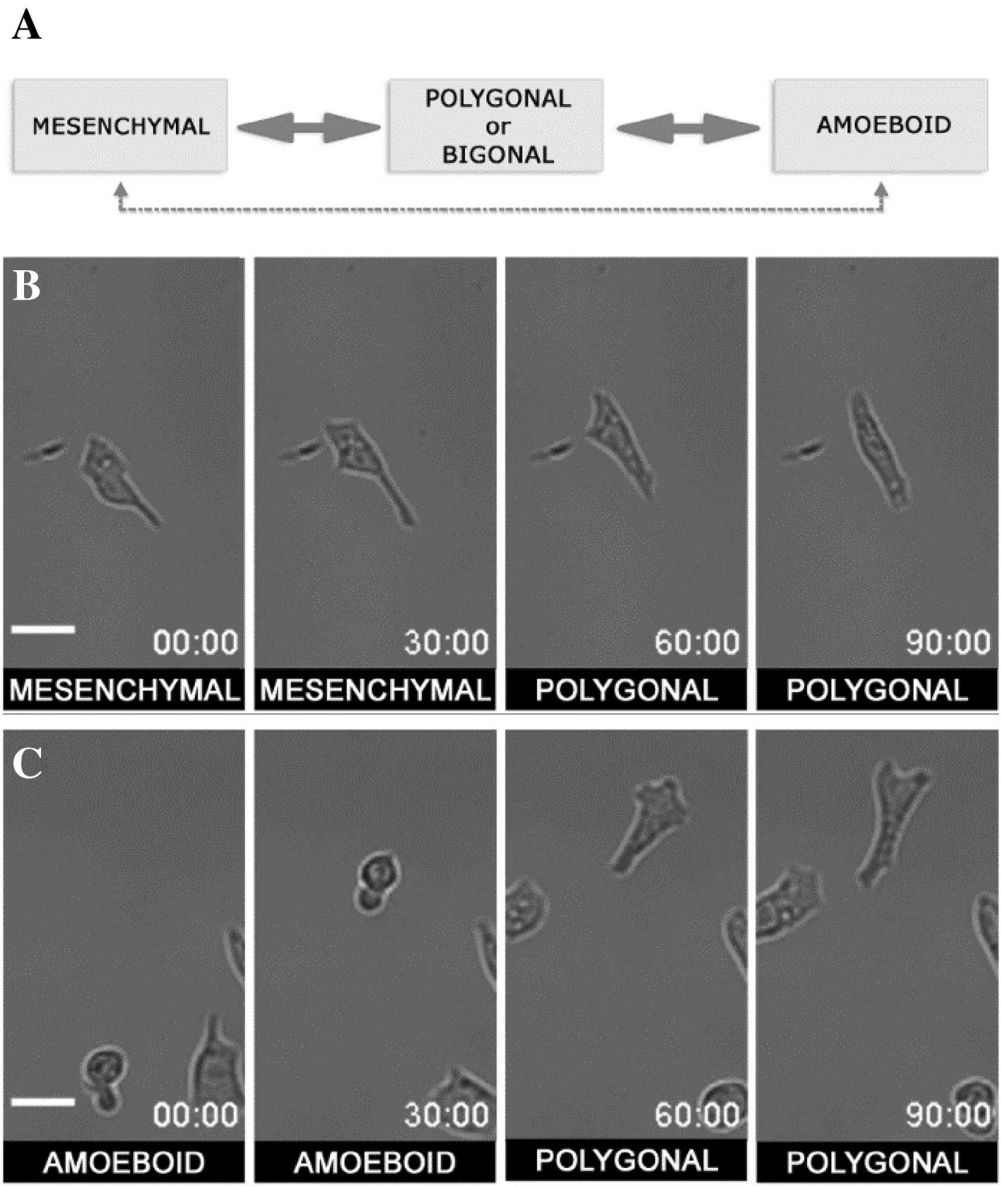
(**A**) Subpopulations transitions observed in migrating cells of WC256 adherent subline. The main transition path marked with solid arrows starts from mesenchymal and goes through polygonal and bigonal to amoeboid subpopulation. Direct transition between mesenchymal and amoeboid subpopulation (marked with dashed arrows) was observed as well, although very rarely (3% of all observed cells). (**B**) Exemplary transition between mesenchymal and polygonal form. (**C**) Exemplary transition between amoeboid and polygonal form. Time scale in minutes:seconds, scalebar = 25 µm. Dynamic behaviors of the same cells are presented in Supplementary Video S3.

A single cell of the WC256 line may exhibit properties of more than one subpopulation over time. During the 4h observation, subpopulation transitions were seen to occur without any external chemical or physical stimulation. However, not every cell underwent such transitions; cells with persistent morphology always remained. The types of observed transitions are shown in Fig. 2A. Transitions marked by solid arrows were the most common. A standard mesenchymal cell could transition into a polygonal/bigonal cell, while the bigonal and polygonal cells could transition to mesenchymal and amoeboid cells. The amoeboid cells primarily transitioned to polygonal/bigonal cells. Only a few cells performed a “full transition” from mesenchymal to polygonal/bigonal (non-polarized) and then amoeboid (or in reverse). There was also a very small fraction (3 out of 100) of cells that performed a direct transition between the mesenchymal and amoeboid subpopulations. Cells performing the two main transitions (mesenchymal ↔ polygonal/bigonal; polygonal/ bigonal ↔ amoeboid) are shown in Fig. 2B, C and Supplementary Video S3.

### Influence of cellular substrate on subpopulation transitions of the WC256 line

**Figure 3:**
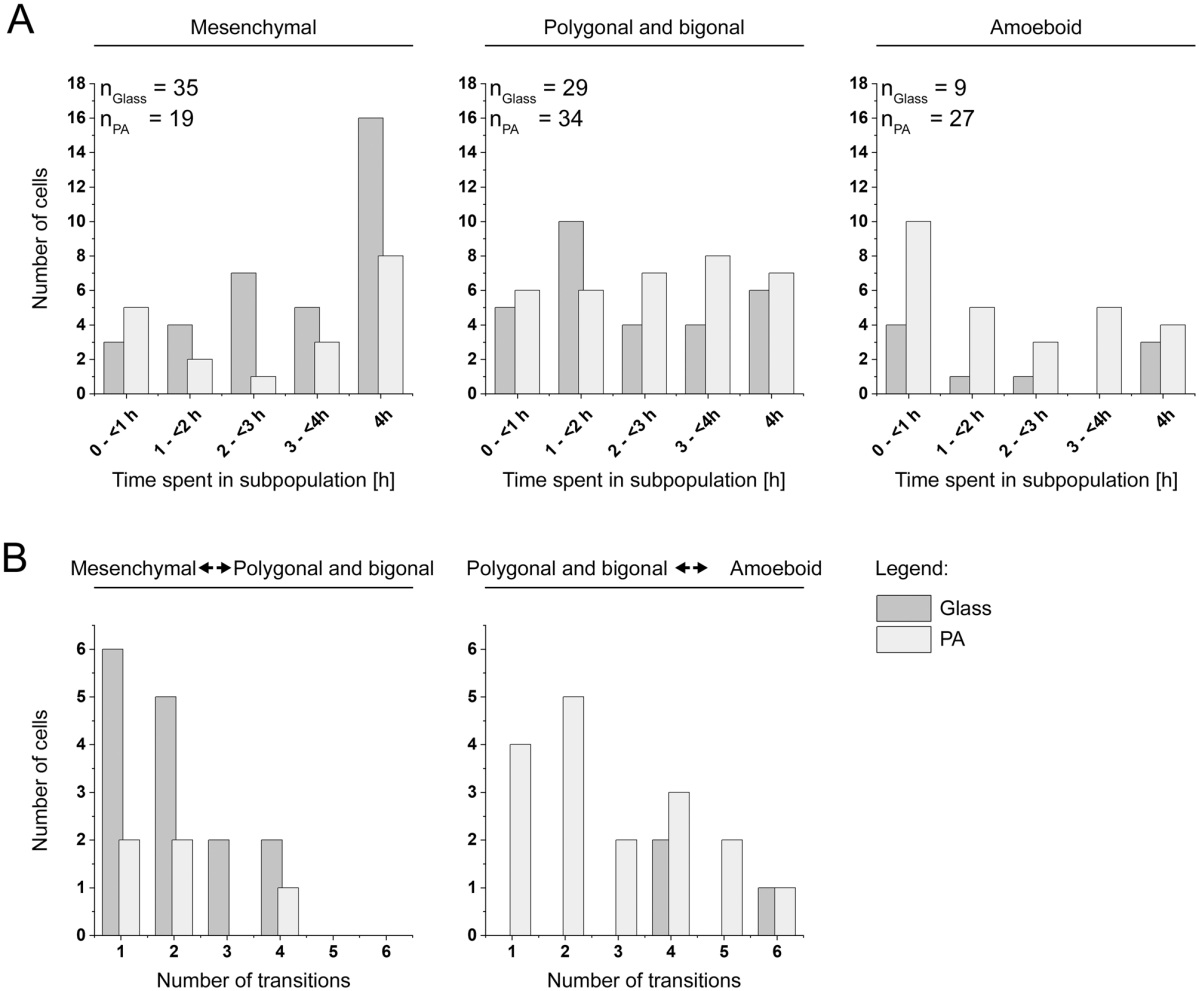
The influence of the mechanical properties of substrates on the dynamics of WC256 cells; (**A**) histograms of time spent in each subpopulation by WC256 cells; (**B**) histograms of number of transitions performed by dynamic cells, presented for two most common transitions (mesenchymal ↔ polygonal/bigonal; polygonal/bigonal ↔ amoeboid).

**Figure 4:**
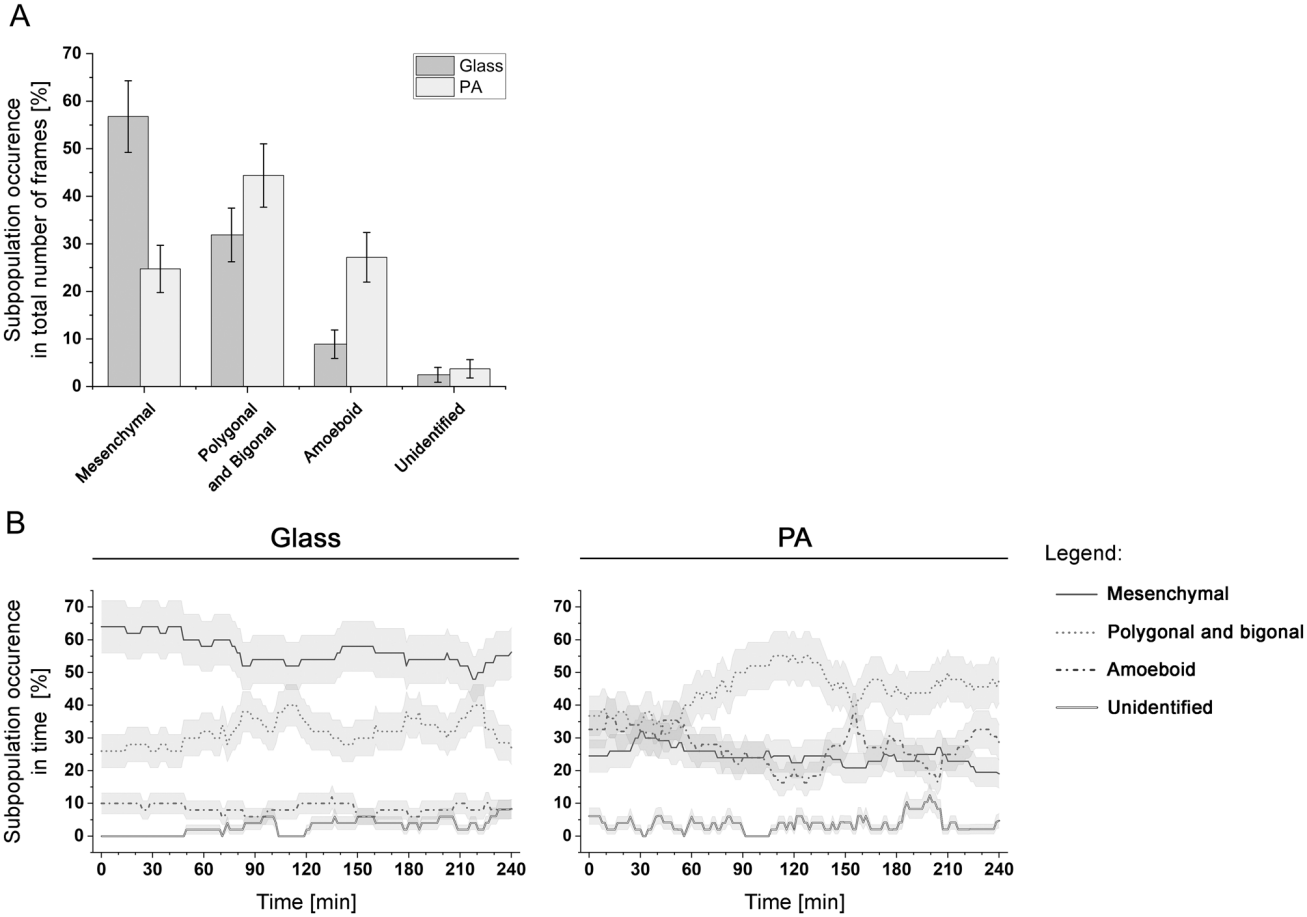
(**A**) The occurrence of different subpopulations in WC256 adherent subline plated on glass or elastic PA substrate. Each bar represents the share of frames of each subpopulation in the total number of registered frames; error bars represent the square root of counts. (**B**) Time evolution of total share of each subpopulation; error bars represent the square root of counts.

**Figure 5:**
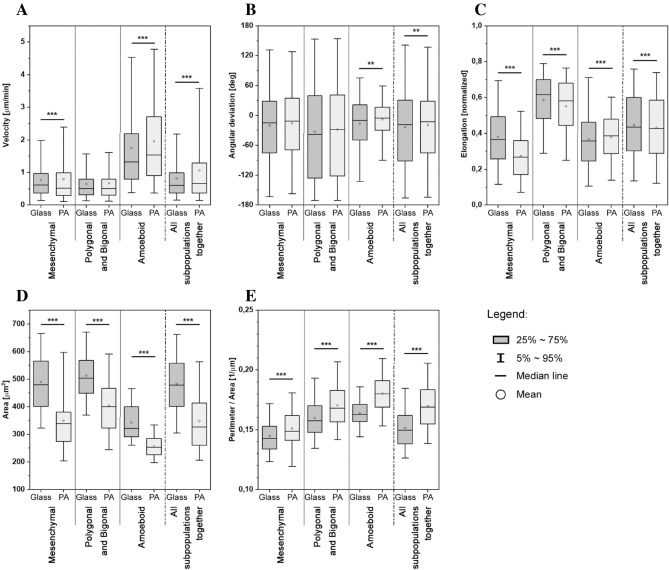
Box plots of velocities, turning angles, elongations and perimeter/area ratios of each WC256 subpopulation, cultured on glass or polyacrylamide (PA) substrate. Number of stars marks the statistical significance calculated by Mann–Whitney test (* for p < 0.05, ** for p < 0.01, *** for p < 0.001).

To analyze the influence of substrate micromechanics on the WC256 line, its impact on the number of cells exhibiting properties of a certain subpopulation or subpopulation transitions was first assessed (Fig. 3). Then, the occurrence of particular subpopulations was calculated throughout the length of the observation (Fig. 4). Finally, the basic biophysical parameters of migration and morphology were analyzed to determine the influence of the applied substrate on WC256 subpopulation behaviors compared to the entire cell line (Fig. 5).

The calculation of morphologies duration in each cell was performed for the entire observation period (4 h). For each substrate, 50 cells from 5 experiments were analyzed and pooled together. The histograms in Fig. 3A demonstrate the time cells spent in specific subpopulations, depending on the cellular substrate. As some observed cells transitioned between subpopulations and, therefore, belonged to two or three subpopulations over time, the total number of cells in Fig. 3A is larger than 50 for each substrate. Fewer cells belonged to the mesenchymal subpopulation on the PA substrate compared to the glass substrate (19 vs. 35 cells, respectively). For both substrates, long mesenchymal states (4 h) were preferred; however, on the PA substrate, there were more cells that belonged to this subpopulation for a shorter period of time (< 1 h). The number of cells in the polygonal/bigonal subpopulation was similar between the PA and glass substrates (34 and 29 cells, respectively). The duration of this subpopulation was distributed evenly throughout the observation period on the PA substrate. On the glass substrate, there was a slight dominance of polygonal/bigonal states that lasted between 1–2 h; however, the remaining time intervals were also distributed evenly. The largest difference between the substrates in terms of the number of cells was in the amoeboid subpopulation; there were 3 times fewer cells on the glass substrate. Cells seeded on the PA substrate primarily spent a very short amount of time in the amoeboid subpopulation, although long occurrences (4 h) were also observed. Among the cells seeded on the glass substrate, a similar number of cells spent a very short (< 1 h) and long (4 h) time in the amoeboid subpopulation. Intermediate times were either rarely observed or not observed at all. However, it is hard to determine whether these differences were caused by the cellular substrate or due to a low occurrence of the amoeboid subpopulation on the glass substrate.

Further analysis determined how many transitions could be performed during the entire 4h experiment for the two most common subpopulation transitions (Fig. 3B). The highest number of transitions performed by a single cell was observed between polygonal/bigonal and amoeboid state (up to 6 transitions), however most cells transitioned between those subpopulations only once or twice. The maximum number of transitions between mesenchymal and polygonal/bigonal subpopulation was 4, nevertheless, most of the cells performed such action only once or twice as well. However, the influence of substrate properties on the number of transitions remains still unclear, as the number of most common transitions differed between the glass and PA substrates. Therefore, it was not possible to determine whether the visible differences shown in Fig. 3B resulted due to the difference between substrates or due to different occurrences of this phenomena. Interestingly, full transitions from the mesenchymal to the amoeboid subpopulation occurred spontaneously without additional stimulation. In total, 4 cells (2 cells per substrate) performed this transition by passing through the polygonal/bigonal subpopulation, and 3 cells (2 on the glass substrate and 1 on the PA substrate) accomplished this transition directly.

### Influence of cellular substrate on subpopulation distribution of the WC256 line

The occurrence of all subpopulations during the entire observation time was calculated. All frames of cells exhibiting properties of particular subpopulations were summed and divided by the number of registered time frames. There were several time frames (< 5% in total) that had unidentified morphology. The occurrence of each subpopulation is shown in Fig. 4A. The PA substrate had a clear impact on the appearance of WC256 subpopulations. On the glass substrate, the mesenchymal subpopulation was observed in over 55% of the time frames, while it was observed in less than 25% of PA substrate time frames. On the PA substrate, an increase in the occurrence of the polygonal/bigonal (from 31.87% on glass to 44.38% on PA) and amoeboid (from 8.89% on glass to 23.17% on PA) subpopulations was observed. Substrate properties had an impact on the number of time frames that fit a defined morphology and performed transitions between subpopulations, as well as on subpopulation occurrence.

Figure 4B gives a better insight into the dynamics of WC256 subpopulations in time. For the cells seeded on glass substrate, the slight decrease of mesenchymal subpopulation occurrence paired with the slight increase of polygonal and bigonal cells over the course of experiment can be observed. It is in line with the fact, that transition between those two populations were most commonly observed on glass substrate and is confirmed by the significant anticorrelation of these subpopulations in time (Pearson’s r = −0.8616*, p < 0.0001) as presented in Table 1. At the same time, the occurrences of amoeboid and undefined cells remained low and stable. On the other hand, the occurrence of mesenchymal subpopulation on PA substrate does not change much over time. However, the temporal fluctuations of polygonal or bigonal as well as amoeboid subpopulations can be observed. Those subpopulations are significantly anticorrelated (Pearson’s r = −0.8274*; p < 0.0001), which also confirms the fact, that transitions between those two subpopulations were observed most frequently on PA substrates. It is important to mention, that very low p-values come from the transitions between subpopulations, which means that they should be treated as dependent variables. In Table 1 we can also observe other statistically significant correlations occurring on glass substrate, however due to the low occurrence of amoeboid subpopulation, this result should not be considered reliable. Similarly, the correlation between mesenchymal and polygonal or bigonal cells on polyacrylamide substrate is statistically significant, however the Pearson’s r = 0.5151 shows that correlation between those two subpopulations is definitely weaker than between polygonal or bigonal and amoeboid cells described above.

**Table 1:** Pearson’s r correlations for time-dependence of time evolution of total share of each subpopulation.

Glass			PA		
	Mesenchymal	Polygonal and bigonal		Mesenchymal	Polygonal and bigonal
Polygonal and bigonal	−0.8616* (p < 0.0001)	–	Polygonal and bigonal	−0.5151* (p < 0.0001)	–
Amoeboid	0.3330* (p < 0.0001)	−0.4538* (p < 0.0001)	Amoeboid	0.1249 (p = 0.1145)	−0.8274* (p < 0.0001)

### Influence of cellular substrate on migration and morphology of WC256 subpopulations

As the application of 40 kPa substrate was found to impact the occurrence of subpopulations, a consecutive step was made to examine whether it also had an impact on cellular shape and migration by comparing biophysical parameters, such as velocities, turning angles, cellular elongations, areas and perimeter/area ratios. These parameters were analyzed for each subpopulation separately, as well as for the entire WC256 population. 

As shown in Table 2 and Fig. 5A, WC256 subpopulations have different velocities. The median velocity of the entire cell line was mostly influenced by the predominant share of mesenchymal and polygonal/bigonal cells. These cells exhibited similar velocities, despite mesenchymal cells typically being migratory polarized cells, while polygonal/bigonal cells are more chaotic, sometimes exhibiting Lévy flights. The greatest impact of applied substrate was observed in amoeboid cells. Although the median velocity of amoeboid cells was moderately affected (increased from 1.325 µm/min to 1.536 µm/min), the difference between the 3rd quartile was 0.531 µm/min, indicating that amoeboid cells migrated faster on the PA substrate. The velocities of polygonal/bigonal cells were not affected by substrate micromechanics, which was expected due to their chaotic movement. Mesenchymal cells migrated on the PA substrate slightly slower than on the glass substrate (Δ = 0.096 µm/min). The median velocity for the entire WC256 population was almost unaffected by 40 kPa substrate; however, a significant increase was observed in the 3rd quartile (from 0.988 µm/min to 1.287 µm/min). This indicates that the increases in amoeboid subpopulation velocity paired and occurrence had a significant impact on the velocity of the entire WC256 population. The significance of a velocity difference between glass and PA substrate registered for the mesenchymal cells and the entire population could be due to the large number of measurements taken rather than a practical implication of this result.

**Table 2:** Quantitative descriptors, namely velocity, turning angles, elongations, area and perimeter/area ratio of each subpopulation compared to the whole population of WC256 cells.

Parameter	Subpopulation	Substrate	Median	Q1	Q3	IQR	Statistical difference
Velocity (µm/min)	Mesenchymal	Glass	0.614	0.372	0.963	0.591	***
		PA	0.518	0.289	0.989	0.700	
	Polygonal and bigonal	Glass	0.509	0.311	0.795	0.484	*–*
		PA	0.507	0.306	0.797	0.492	
	Amoeboid	Glass	1.325	0.795	2.186	1.391	***
		PA	1.536	0.899	2.717	1.818	
	All subpopulations together	Glass	0.607	0.364	0.988	0.624	***
		PA	0.655	0.365	1.287	0.922	

Turning angles (°)	Mesenchymal	Glass	−15.4	−75.7	28.6	104.2	–
		PA	−11.8	−68.9	34.4	103.3	
	Polygonal and bigonal	Glass	−37.9	−126.1	39.8	166.0	–
		PA	−28.2	−121.3	41.2	162.5	
	Amoeboid	Glass	−10.4	−49.1	21.7	70.9	**
		PA	−5.3	−29.6	16.6	46.2	
	All subpopulations together	Glass	−18.6	−91.2	30.2	121.4	**
		PA	−15.4	−75.7	28.6	104.2	
Elongation (normalized)	Mesenchymal	Glass	0.365	0.258	0.493	0.235	***
		PA	0.267	0.169	0.359	0.190	
	Polygonal and bigonal	Glass	0.615	0.484	0.700	0.216	***
		PA	0.580	0.445	0.681	0.236	
	Amoeboid	Glass	0.357	0.245	0.462	0.217	***
		PA	0.385	0.287	0.480	0.193	
	All subpopulations together	Glass	0.436	0.302	0.601	0.299	***
		PA	0.427	0.288	0.585	0.297	

Area (µm^2^)	Mesenchymal	Glass	478.9	401.5	566.0	164.5	***
		PA	338.3	274.6	381.1	106.5	
	Polygonal and bigonal	Glass	503.9	448.9	568.2	119.3	***
		PA	399.3	323.1	466.3	143.3	
	Amoeboid	Glass	321.4	290.9	399.3	108.4	***
		PA	252.8	226.1	284.9	58.8	
	All subpopulations together	Glass	478.3	401.0	558.4	157.4	***
		PA	326.3	259.9	414.0	154.2	

Perimeter/area (1/µm^2^)	Mesenchymal	Glass	0.142	0.134	0.153	0.019	***
		PA	0.149	0.141	0.162	0.021	
	Polygonal and bigonal	Glass	0.158	0.148	0.170	0.022	***
		PA	0.168	0.157	0.183	0.026	
	Amoeboid	Glass	0.163	0.157	0.171	0.014	***
		PA	0.180	0.169	0.191	0.022	
	All subpopulations together	Glass	0.150	0.138	0.162	0.024	***
		PA	0.169	0.155	0.184	0.029	

Number of stars marks the statistical significance calculated by Mann–Whitney test (– for no difference * for p < 0.05, ** for p < 0.01, *** for p < 0.001).

The widest distribution of turning angles on both substrates (> 160°) was observed in polygonal/bigonal cells, as shown in Table 2 and Fig. 5B. This was expected due to their chaotic movement and was not altered by the substrate type. The mesenchymal subpopulation was characterized by a narrower distribution of turning angles on both substrates (< 105°), indicating more directed movement that was not altered by application of 40 kPa substrate. It only impacted the distribution of turning angles in the amoeboid subpopulation, which migrated more straightly on the PA substrate (IQRs on the glass and PA substrates were 70.9 and 46.2, respectively). The distribution width for the entire WC256 population was 18 degrees narrower on the PA substrate; which could be due to the higher occurrence of the amoeboid subpopulation on this substrate. However, this statistical significance was rather caused again by the large sample size rather than practical differences in turning angles.

The morphologies of WC256 subpopulations were remarkably different from each other (Table 2, Fig. 5C–E). Mesenchymal and amoeboid cells seeded on the glass substrate had similar elongation factors (0.365 and 0.357, respectively); however, polygonal/bigonal cells were the most elongated ones. The elongation of the mesenchymal subpopulation was visibly affected on 40 kPa substrate, appearing significantly rounder there. The elongation of the polygonal/bigonal and amoeboid subpopulations was significantly different between the glass and PA substrates; however, it appears that this difference was again caused by the large sample size. The impact of substrate micromechanics on the elongation of the entire WC256 population was small (only a 0.009 difference between substrates), which poorly reflects the variety of subpopulations morphologies. Cells observed on elastic substrate had also significantly smaller area (Table 2 and Fig. 5D), which is applicable to all subpopulations. The impact of substrate type on cell area was however the highest among mesenchymal cells (478.9 µm^2^ on glass compared to 338.3 µm^2^ on elastic substrate). In contrary, the ratio of cell perimeter to its area (Table 2 and Fig. 5E) increased on elastic substrates, with the greatest increase for amoeboid subpopulation (from 0.163 to 0.180 1/µm). All three parameters of cell shape described here show that cellular shapes of WC256 cells significantly changed, when polyacrylamide elastic substrates were applied.

## Discussion

Current research indicates that heterogeneity is an inherent property of biological populations. This carries profound consequences for the biological sciences, as the fact that biological objects (in this case, cells) differ from one another has to be considered when analyzing experimental data. The current study has characterized the time evolution of morphology and migration parameters in adherent WC256 sublines for the first time. Previous studies^41, 43^ have characterized the entire WC256 population, including adherent and non-adherent sublines. Such studies demonstrated the complexity of WC256 cell morphology involving mesenchymal, polygonal/bigonal, and amoeboid (blebbing) cells. In the current study, based on time-lapse observations, cellular migration strategy and morphology were considered as two interrelated dynamic features that influence cell behavior. This approach enabled the construction of behavioral, dynamic criteria for the classification of WC256 subpopulations. This improves upon previous classifications, which were based on static images at specific time points. Previous classifications of WC256 subtypes are easily comparable with the approach presented in Fig. 1 and Supplementary Video S1.

The current study found a significantly larger number of mesenchymal (lamellipodia-creating) cells compared to polygonal/bigonal (nonpolar) cells on the glass substrate. This is in clear opposition to previous findings^41^ and is thought to be due to several factors, including the way cellular subtypes were classified. During static analysis, mesenchymal cells could be classified as nonpolar due to their heterogenous morphology, as lamellipodia may not be clearly distinguishable (Supplementary Videos S1 and S2). Therefore, the analysis of time-lapse sequences is required to properly classify WC256 cell types in bright-field microscopy. The reversed proportion between mesenchymal and nonpolar subpopulations might also be a result of fibronectin coating, which was not present in previous work^41^ but can significantly influence cell adhesion and morphology^44, 45^.

The current analysis of time-lapse experiments revealed that the morphology and migration strategy of cells were unstable. WC256 cells were found to be capable of transitioning from one subpopulation to another, with 40% of cells seeded on the glass substrate exhibiting spontaneous transitions that occurred during the experiment without any external physical or chemical stimulation. The transitions observed were dynamic and reversible and could occur several times in a single cell. Current literature presents numerous examples of cells transitioning from mesenchymal to amoeboid (MAT) subpopulations and from amoeboid to mesenchymal (AMT) subpopulations. However, these examples were triggered by various physical, chemical, and biological factors, including the local ECM environment^46^, surface coating and confinement introduction^47^, perturbation of actin polymerization and actomyosin contractility^43, 48^, and integrin receptor blocking^49^. Previous research has stated that a more pronounced shift between ameboid and mesenchymal morphology requires long-term culture under specified conditions and is likely due to epigenetic changes^41^. The most common transitions observed in the current study occurred between mesenchymal and polygonal/bigonal subpopulations, as well as between the polygonal/bigonal and amoeboid subpopulations. Even if they did not undergo MAT/AMT transitions, the observed cells could change their morphology significantly by losing or establishing polarization. A small number of cells underwent a full MAT/AMT transition, passing through the intermediate polygonal/bigonal state. To the authors’ knowledge, there are no other reports of transitions similar to these. The spontaneous nature of these transitions in any 2D in vitro culture has also not been previously observed. This may be due to the long migration observations used in the current study, which were densely sampled. The time-lapse observation of cancerous cells used in the current study may be the key to improving further research by providing greater insights into cellular functioning.

The velocity of mesenchymal subpopulation seeded on the glass substrate was similar to the velocity of “lamellipodia-forming cells” distinguished in previous work^42^, while the amoeboid subpopulation was significantly slower. Such differences may stem from the fact that in previous studies, amoeboid cells were directly derived from a non-adherent subline and had weak substrate adhesion that promoted effective migration due to bleb formation. In contrast, the amoeboid cells in the current study were part of an adherent subline and were present in approximately 10% of all observed glass substrate frames. The epigenetic differences between non-adherent and adherent sublines and, consequently, differences in adhesion levels might explain this velocity difference. It also highlights the lack of difference between well-spread cells that are similar to each other in the current study and previous studies.

In the current study, mechanical microenvironment impacted WC256 cells in several ways, one of which was the distribution of subpopulations. The glass substrate had more mesenchymal cells but fewer amoeboid cells compared to the PA substrate. Only the polygonal/bigonal cells were similarly abundant on both substrates. The differences in occurrence and duration of subpopulations and their durations were clearly visible. On the glass substrate, mesenchymal cells were the predominant subpopulation, while on the PA substrate, it was the polygonal/bigonal subpopulation. The amoeboid subpopulation had a low presence on the glass substrate, being more abundant on the PA substrate. This ‘shift’ from a well-spread mesenchymal to a poorly-spread amoeboid subpopulation may be due to the possible presence of obstacles in substrate-cell interactions on the PA substrate, which is in line with previous models that state that softer substrates promote blebbing and pseudopodial morphology over mesenchymal morphology^50^. The influence of elastic substrates on cell morphology and migration has been widely discussed in previous literature^15, 30, 51–54^, as well as their role in tumor progression^19, 55^. Many different factors have been discussed within the context of tumor heterogeneity, including epigenetic and genetic alterations^56^. The current work provides important evidence that substrate properties, especially mechanical ones, can significantly impact the distribution of cell subpopulations and, therefore, cancer heterogeneity. This result is significant within the context of cancer development, as the mode of migration is a crucial aspect of metastasis.

The ratio of mesenchymal to amoeboid cells within a population, as well as the plasticity of transitions between them, can determine the probability of cancer dissemination. A significant change in subpopulation distribution, as observed in the current study, can significantly impact the results of migratory experiments due to the involvement of a completely different set of signaling pathways^57^. This becomes particularly important when considering potential antimetastatic agents. One of the most important results in the current study was the observed shift towards the amoeboid mode of migration on the PA substrate. The significance of the amoeboid mode of cancer cell migration was recently summarized^57,58^. It is connected to an improved ability of cancer cells to disseminate and cancer cell stemness. There is indirect evidence that the dependence of MDA-MB-231 and A375M2 cell lines (human breast cancer and melanoma cells, respectively) on PKCα amoeboid migration correlates with their invasiveness^59^. Cancer cells that easily change their mode of migration from mesenchymal to amoeboid can avoid proteolytic enzyme inhibitor-based therapy^60^. The ability of the adherent WC256 subline used in the current study to switch from a mesenchymal to amoeboid mode of migration without external stimulation, particularly on the PA substrate, is an important finding due to its potential use in cancer therapy.

Novel computational models predict that particular modes of migration are advantageous within homogeneous conditions of low or high resistance (amoeboid and mesenchymal migration, respectively)^61^. The same models demonstrate that high plasticity of mesenchymal to amoeboid transitions is beneficial in heterogeneous microenvironments, which are typically in vivo^61^. The current study considered direct MAT/AMT transitions, as well as the intermediate stages. It revealed a high plasticity on both substrates, with cells rapidly adapted to them. A mathematical model of cancer cell migration in extreme modes in a maze-like 2D environment revealed an additional advantage of heterogeneity within populations, as the proteolytic activity of mesenchymal cells paved the way for amoeboid cells^62^. In the current study, the PA substrate led to a substantially higher proportion of amoeboid cells and cells that could dynamically change their mode of migration to amoeboid, however there was a small population of mesenchymal cells that could turn into amoeboid. The amoeboid mode of migration was underestimated for a long time due to its rare occurrence and difficult characteristics on rigid 2D substrates. It has drawn wider attention since migration research has begun using 3D conditions^63^. Although the current study model does not simulate a 3D microenvironment, it is still able to reflect in vivo conditions while maintaining the convenience of 2D in vitro migration models. As tumor heterogeneity is an important factor in cancer diagnosis and treatment^3, 4, 56^, further studies are needed to elucidate the effects of the mechanical interactions between a tumor and its microenvironment.

In addition to subpopulation duration, transition, and distribution, substrate micromechanical properties impacted the morphology and migration parameters of some WC256 subpopulations. Mesenchymal cells were rounder on the PA substrate, but other parameters in this subpopulation were poorly influenced by the type of substrate. No practical differences were observed in polygonal/bigonal cells as their velocity and turning angles did not change; however, they became slightly rounder on PA substrate. In the amoeboid subpopulation, cellular velocity visibly differed, with faster cells seen on the PA substrate. The current data consisted of a large number of samples; therefore, even very small changes appeared statistically significant. Therefore, even in parameters where statistically significant differences were reported (e.g., mesenchymal cell velocity, polygonal/bigonal, and amoeboid cell elongation), the practical implications in some cases could be minimal. However, the elastic substrate did impact the subpopulations differently in the context of migration and morphology parameters. The existing literature demonstrates that cell properties, for example, cell growth, can depend on substrate rigidity^17^, although this impact differs for particular cell lines. The current study clearly demonstrates that even in one cell line, distinct subpopulations are present and respond differently to the mechanical properties of the microenvironment.

The current data also highlights the significance of unitary analyses of heterogenous cell lines. Across all analyzed parameters of cell migration and morphology, the results obtained across the entire WC256 population differed from those obtained for each subpopulation. It is crucial that heterogeneous cell lines are analyzed in the context of their subpopulations, as averaging parameters may significantly change the final results^2^.

Two different cellular substrates were employed in these studies—glass and polyacrylamide-based hydrogel with Young modulus of 40 kPa. It is known that the mechanical properties of the microenvironment significantly impact many biological processes in physiological and cancerous cell lines, including stem cells. One of them is substrate elasticity which can alter cell proliferation^17, 51, 55, 64^, spreading^30, 64^, morphology^15, 51, 52^, F-actin organization^30, 54, 65^, migration velocity^30, 53^ and directionality^53, 54^. However, other substrate properties such as substrate adhesiveness, porosity, ECM patterning or dimensionality can influence the cell behavior. In this study, the mechanism of ECM binding on glass and PA substrates differed, so even if the similar concentration of protein solution during biofunctionalization of the substrates was provided, the resulting density of extracellular matrix proteins might differ. Current literature shows, that ligand density interplays with substrate elasticity, resulting in the modified cell spreading^44^. Another aspect in which glass and PA substrate differ is the porosity. While generally glass is smooth, the PA gels exhibit pores that in substrates that are softer (30 kPa) than the one used in this study, reach up to 45 nm^34^. However, the porosity does not need to impact cellular functioning in subject of their differentiation or magnitude of traction forces, while regardless of the porosity, those processes were regulated predominantly by the substrate elasticity^34^. Taking all of this into consideration, we expect the substrate elasticity to be the major factor causing changes in the dynamic heterogeneity of WC256 Walker carcinosarcoma cells. Nonetheless, further investigation of the interplay of different aspects of substrate micromechanics on cellular heterogeneity is necessary to give a better insight into mechanisms of cancer biology.

## Limitations and further issues

The current study involved the 4-h observation of transitions between different subpopulations of an adherent WC256 subline. To find prospective longer transitions and investigate this uncommon phenomenon in greater depth, future studies should extend the observation time while maintaining a similar degree of sampling to capture all significant information. In this study, the 40 kPa substrate was used to mechanically mimic breast parenchymal tissue, which has elasticity between 30 and 50 kPa^66^ and to create a biomimetic microenvironment for the rat breast carcinosarcoma cells. However, the breast also consists of fat tissue that has a Young modulus of approximately 7 kPa, and the elasticity of cancerous breast tissues can vary from 20 to 100 kPa depending on their malignancy^66, 67^. Furthermore, the composition and concentration of the ECM is not typically even within the whole breast. The dimensionality of the experimental setup could also have influenced the results, as cells in 2D, 3D, and sandwich-like environments can exhibit different migration modes^50, 57^. Therefore, the comparison of cellular transitions and morphologies on softer and stiffer substrates, combined with different types of ECM and substrate dimensionality, will provide deeper insights into the biophysical and biochemical mechanisms of this phenomenon. However, future studies will require the invention of new quantitative methods for analyzing cellular morphology and migration properties since such datasets will contain tens to hundreds of thousands of frames that each need to be assigned to their proper subpopulation.

## Materials and methods

### Cell culture

Walker carcinosarcoma cells were obtained from Professor H. Keller (University of Bern, Bern, Switzerland). The adherent subline of WC256 cells was derived from the initially non-adherent cells grown in a suspension by Dr. Jolanta Sroka^41^. The WC256 cells were cultured in an RPMI-1640 medium (EuroClone) supplemented with 5% fetal bovine serum (Gibco), 100 IU/mL penicillin, and 10 μg/mL streptomycin (Sigma-Aldrich). Cells were maintained in a humidified 37 °C incubator with 5% CO_2_^42^.

### Substrate preparation

Two types of substrates were used: glass-bottom dishes for control samples and elastic PA substrates for experimental samples. The dishes were silanized via treatment with an acetic acid solution of 3-(trimethoxysilyl) propyl methacrylate (Sigma-Aldrich) and 96% ethyl alcohol in a 1:1:14 proportion for 20 min. They were subsequently rinsed three times using 96% ethyl alcohol^68^. These procedures were performed to increase the affinity of the PA substrate for glass^69, 70^. The PA substrates with a Young modulus of E = 40 kPa were prepared according to standard procedure, using a 40% aqueous solution of acrylamide (Bio-Rad), a 2% aqueous solution of Bis-Acrylamide (Bio-Rad), and red fluorescent beads (Invitrogen) diluted in a 10 mM aqueous solution of HEPES (Sigma-Aldrich)^11, 25, 69, 70^. As the WC256 cells came from rat carcinosarcoma, the 40 kPa elasticity was chosen to mimic the elasticity of breast parenchymal tissue^66^. The mixture was polymerized using 10% ammonium persulfate (Bio-Rad) and tetramethylethylenediamine (BioShop) in ratios of 1/200 and 1/2000 to the total volume of the mixture. Then, 23 μl of the solution was applied to the silanized glass bottom plates. Each drop was covered with an 18 mm (diameter) coverslip. Polymerized substrates were activated by applying the Sulfo-SANPAH solution (Thermo Scientific) and 5 min of ultraviolet light. The substrates were then washed three times with sterile PBS. The hydrogels were incubated with a 15 μg/ml fibronectin solution at 4 °C for 12 h^69, 70^. The glass-bottom dishes used in the control experiments were incubated with the same fibronectin solution.

### Time-lapse registration of cell migration

WC256 cell migration was recorded using the time-lapse method and an inverted Axio Observer.Z1 microscope (Zeiss), AxioCam camera (Zeiss), and a Plan Apochromat 10×/0.45 objective (Zeiss). Data acquisition was carried out for the duration of 4-h observation at 90-s intervals. Cell migration registration started 1 h after seeding (30 min after cell adhesion was observed). A temperature of 37 °C and a CO_2_ concentration of 5% were maintained during image acquisition. The definite focus component (Zeiss) was used to maintain the proper focal plane.

### Image analysis

During the image analysis, 50 cells from five control and five experimental samples were analyzed. Binary masks were obtained from the brightfield images. Cells with sharp boundaries and regular shapes were analyzed automatically using cell migration software that produced binary masks^71^. Cells that were not appropriately identified by the software were analyzed manually using the ROI Tracker plugin^72^ in the ImageJ software. The perimeters were converted to binary masks using ImageJ.

**Figure 6:**
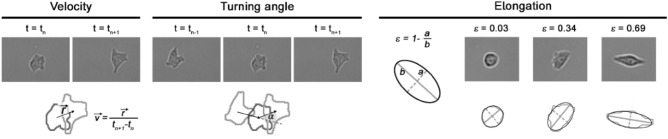
Selected quantitative parameters describing cell migration and morphology.

The binary masks were analyzed using a custom script in MATLAB. From each binary mask, the centroid and elongation were calculated. The velocity in each frame was calculated by dividing the displacement in the current (n) and following (n + 1) frames by the time interval. Turning angles were calculated as the angle between two subsequent displacement vectors (i.e., between frames n − 1 → n and n → n + 1). Elongation was defined as ε = 1 − (a/b), whereby a and b represent the major and minor axis of the ellipse fitted to binary shape, respectively (ε = 0 for circular shapes and ε = 1 for infinitely elongated ellipse). Cell area and perimeter were obtained from binary masks and recalculated to the pixel size. Quantitative descriptors of migration and morphology are presented in Fig. 6.

### Inclusion criteria

This study aimed to analyze single migrating cells that did not visibly interact with each other. Therefore, only cells that met certain criteria were analyzed: (1) the cell had to be an individual; (2) the analyzed cell could not be in contact with another cell; and (3) the cell could not be undergoing division in current or neighboring frames. In most cases, cell division starts with cell rounding, followed by spreading on the substrate. However, before and after this process, WC256 cells maintained a similar morphology. Thus, only those frames in which cells were not rounded or were already spread after the division process were analyzed. Frames where a cell was interacting with another cell either by direct collision or visible changes in shape or migration direction that occurred only near a neighboring cell were excluded from the analysis.

### Subpopulations analysis

Different subpopulations were distinguished similarly to previous work^41^, with mesenchymal, polygonal/bigonal, and amoeboid classifications used. The current study focused on the qualitative identification of cell types as robust and universal quantitative descriptors for the discrimination of cell subpopulations do not exist. To be qualitatively identified as mesenchymal, cells had to meet several criteria: the one-way expansion of the cell border had to result in the directional displacement of the cell centroid in a short timeframe (several image frames); movement had to involve either a keratinocyte-like gliding motion or repeated cycles of protrusion and attachment of the cell leading edge, coordinated with the detachment and retraction of the cell rear^57, 73, 74^; and a similar shape (mostly well spread) had to be maintained over time. To be qualitatively identified as polygonal/bigonal, cells had to meet different criteria: expansion of the cell border had to occur in more than one direction (cell stretching) and result in the non-directional displacement of the cell centroid in a short timeframe; there could only be limited moments in which the cell tore off from one adhesion site, which had to result in the displacement of the cell centroid; and similar morphology (mostly well spread) had to be maintained over time. To be qualitatively identified as amoeboid cells, several criteria had to be met: the one-way expansion of the cell border had to result in the directional displacement of the cell centroid in a short timeframe; they had to have an unstable shape (mostly poorly spread) with small dynamic protrusions (pseudopodial cells); or migration had to occur via spherical protrusions on the leading edge (blebbing cells)^43, 47, 57^.

The assignment of subpopulations was conducted within the context of cell dynamics rather than using a simple analysis of cell shape in a given frame. For each frame, the 2 previous and 2 ensuing frames were compared to determine cell behavior. This enabled the observation of membrane extension direction (uni- or multidirectional), which was an important criterion of subpopulation classification. Frames that were hard to classify using the criteria were designated as an “unidentified” subpopulation. This subpopulation was excluded from further analysis of migration and shape parameters.

### Numerical and statistical analysis

Statistical analysis and graph creation were done using the OriginPro 2018 software. Most of data distributions were not normal (D'Agostino' K-squared test), only the Perimeter/Area ratio in Mesenchymal subpopulation met the normality criteria. Therefore, the median and interquartile ranges (IQRs) were used to describe the data and Mann–Whitney test was performed to analyze statistical differences. Widths of distributions are described by IQRs, which are the differences between the 3rd and 1st quartiles (IQR = Q3 – Q1).

The histograms presented in Fig. 3A, B were constructed from the data pooled from all experiments for each substrate. The duration of each subpopulation, presented in Fig. 3A, was calculated as the total time in which a cell belonged to a specific subpopulation. Therefore, if a cell transitioned to a different subpopulation and then transitioned back to the initial subpopulation, the duration of the cell in the initial subpopulation was the summed time it spent in that subpopulation (before and after transitioning). The number of transitions, as shown in Fig. 3B, presents the number of times in which cells transitioned from one subpopulation to another. The subpopulation occurrence, as shown in Fig. 4, was calculated by dividing the number of frames collected for each specific subpopulation across all experiments by the total number of frames collected for each substrate; the error bars presents the square root of counts. The biophysical parameters of cell migration and morphology, as shown in Fig. 5A–E, represent the distribution of all frames observed that were assigned to a specific subpopulation or the entire WC256 population.

### Supplementary Information

Supplementary Legends.

Supplementary Video 1.

Supplementary Video 2.

Supplementary Video 3.

## Data Availability

The datasets generated during and/or analyzed during the current study are available from the corresponding author on reasonable request.

## References

[1] Welch DR (2016). Tumor heterogeneity—A “contemporary concept” founded on historical insights and predictions. Cancer Res..

[2] Altschuler SJ, Wu LF (2010). Cellular heterogeneity: Do differences make a difference?. Cell.

[3] Marusyk A, Janiszewska M, Polyak K (2020). Intratumor heterogeneity: The Rosetta stone of therapy resistance. Cancer Cell.

[4] Flashner-Abramson E, Vasudevan S, Adejumobi IA (2019). Decoding cancer heterogeneity: Studying patient-specific signaling signatures towards personalized cancer therapy. Theranostics.

[5] Dagogo-Jack I, Shaw AT (2018). Tumour heterogeneity and resistance to cancer therapies. Nat. Rev. Clin. Oncol..

[6] Lim Z-F, Ma PC (2019). Emerging insights of tumor heterogeneity and drug resistance mechanisms in lung cancer targeted therapy. J. Hematol. Oncol..

[7] Findlay JM, Castro-Giner F, Makino S (2016). Differential clonal evolution in oesophageal cancers in response to neo-adjuvant chemotherapy. Nat. Commun..

[8] Gallaher JA, Enriquez-Navas PM, Luddy KA (2018). Spatial heterogeneity and evolutionary dynamics modulate time to recurrence in continuous and adaptive cancer therapies. Cancer Res..

[9] Park CC, Bissell MJ, Barcellos-Hoff MH (2000). The influence of the microenvironment on the malignant phenotype. Mol. Med. Today.

[10] Ahmed F, Haass NK (2018). Microenvironment-driven dynamic heterogeneity and phenotypic plasticity as a mechanism of melanoma therapy resistance. Front. Oncol..

[11] Tse JR, Engler AJ (2010). Preparation of hydrogel substrates with tunable mechanical properties. Curr. Protoc. Cell Biol..

[12] McKee CT, Last JA, Russell P, Murphy CJ (2011). Indentation versus tensile measurements of young’s modulus for soft biological tissues. Tissue Eng. Part B Rev..

[13] Engler AJ, Richert L, Wong JY (2004). Surface probe measurements of the elasticity of sectioned tissue, thin gels and polyelectrolyte multilayer films: Correlations between substrate stiffness and cell adhesion. Surf. Sci..

[14] Georges PC, Janmey PA (2005). Cell type-specific response to growth on soft materials. J. Appl. Physiol..

[15] Peyton SR, Ghajar CM, Khatiwala CB, Putnam AJ (2007). The emergence of ECM mechanics and cytoskeletal tension as important regulators of cell function. Cell Biochem. Biophys..

[16] Chen CS (2008). Mechanotransduction—A field pulling together?. J. Cell Sci..

[17] Tilghman RW, Cowan CR, Mih JD (2010). Matrix rigidity regulates cancer cell growth and cellular phenotype. PLoS ONE.

[18] Katira P, Zaman MH, Bonnecaze RT (2012). How Changes in Cell Mechanical Properties Induce Cancerous Behavior. Phys Rev Lett.

[19] Katira P, Bonnecaze RT, Zaman MH (2013). Modeling the mechanics of cancer: Effect of changes in cellular and extra-cellular mechanical properties. Front. Oncol..

[20] Mierke CT (2019). The matrix environmental and cell mechanical properties regulate cell migration and contribute to the invasive phenotype of cancer cells. Rep. Prog. Phys..

[21] Balcerak A, Wakuła M, Trębińska A, Grzybowska EA (2016). Migracja i inwazyjność komórek nowotworowych; rola plastyczności komórek i udział macierzy zewnątrzkomórkowej w tworzeniu przerzutów. Nowotwory J. Oncol..

[22] Emon B, Bauer J, Jain Y (2018). Biophysics of tumor microenvironment and cancer metastasis—A mini review. Comput. Struct. Biotechnol. J..

[23] Kim D-H, Wong PK, Park J (2009). Microengineered platforms for cell mechanobiology. Annu. Rev. Biomed. Eng..

[24] Mofrad MRK (2009). Rheology of the cytoskeleton. Annu. Rev. Fluid Mech..

[25] Pelham RJ, Wang Y (1997). Cell locomotion and focal adhesions are regulated by substrate flexibility. Proc. Natl. Acad. Sci..

[26] Lee KY, Mooney DJ (2001). Hydrogels for tissue engineering. Chem. Rev..

[27] Levental I, Georges PC, Janmey PA (2007). Soft biological materials and their impact on cell function. Soft Matter.

[28] Tang X, Wen Q, Kuhlenschmidt TB (2012). Attenuation of cell mechanosensitivity in colon cancer cells during in vitro metastasis. PLoS ONE.

[29] McKenzie AJ, Hicks SR, Svec KV (2018). The mechanical microenvironment regulates ovarian cancer cell morphology, migration, and spheroid disaggregation. Sci. Rep..

[30] Adlerz KM, Aranda-Espinoza H, Hayenga HN (2016). Substrate elasticity regulates the behavior of human monocyte-derived macrophages. Eur. Biophys. J..

[31] Patel NR, Bole M, Chen C (2012). Cell elasticity determines macrophage function. PLoS ONE.

[32] Boothe SD, Myers JD, Pok S (2016). The effect of substrate stiffness on cardiomyocyte action potentials. Cell Biochem. Biophys..

[33] Trappmann B, Gautrot JE, Connelly JT (2012). Extracellular-matrix tethering regulates stem-cell fate. Nat. Mater..

[34] Wen JH, Vincent LG, Fuhrmann A (2014). Interplay of matrix stiffness and protein tethering in stem cell differentiation. Nat. Mater..

[35] Xu J, Sun M, Tan Y (2017). Effect of matrix stiffness on the proliferation and differentiation of umbilical cord mesenchymal stem cells. Differentiation.

[36] Mceuen CS (1933). The effect of hypophysectomy on the growth of the Walker rat tumour. Br. J. Exp. Pathol..

[37] Chew EC (1976). The fine structure of Walker 256 carcinoma cells. Experientia.

[38] Magro C, Orr FW, Manishen WJ (1985). Adhesion, chemotaxis, and aggregation of Walker carcinosarcoma cells in response to products of resorbing bone. J. Natl. Cancer Inst..

[39] Manishen WJ, Sivananthan K, Orr FW (1986). Resorbing bone stimulates tumor cell growth. A role for the host microenvironment in bone metastasis. Am. J. Pathol..

[40] Simpkins H, Lehman JM, Mazurkiewicz JE, Davis BH (1991). A morphological and phenotypic analysis of Walker 256 cells. Cancer Res..

[41] Sroka J, von Gunten M, Dunn GA, Keller HU (2002). Phenotype modulation in non-adherent and adherent sublines of Walker carcinosarcoma cells: The role of cell-substratum contacts and microtubules in controlling cell shape, locomotion and cytoskeletal structure. Int. J. Biochem. Cell Biol..

[42] Sroka J, Krecioch I, Zimolag E (2016). Lamellipodia and membrane blebs drive efficient electrotactic migration of rat Walker carcinosarcoma cells WC 256. PLoS ONE.

[43] Bergert M, Chandradoss SD, Desai RA, Paluch E (2012). Cell mechanics control rapid transitions between blebs and lamellipodia during migration. Proc. Natl. Acad. Sci..

[44] Engler A, Bacakova L, Newman C (2004). Substrate compliance versus ligand density in cell on gel responses. Biophys. J..

[45] Deeg JA, Louban I, Aydin D (2011). Impact of local versus global ligand density on cellular adhesion. Nano Lett..

[46] Brábek J, Mierke CT, Rösel D (2010). The role of the tissue microenvironment in the regulation of cancer cell motility and invasion. Cell Commun. Signal..

[47] Liu YJ, le Berre M, Lautenschlaeger F (2015). Confinement and low adhesion induce fast amoeboid migration of slow mesenchymal cells. Cell.

[48] Keller HU (2000). Redundancy of lamellipodia in locomoting Walker carcinosarcoma cells. Cell Motil. Cytoskelet..

[49] Carragher NO, Walker SM, Scott Carragher LA (2006). Calpain 2 and Src dependence distinguishes mesenchymal and amoeboid modes of tumour cell invasion: A link to integrin function. Oncogene.

[50] Friedl P, Wolf K (2010). Plasticity of cell migration: A multiscale tuning model. J. Cell Biol..

[51] Skardal A, Mack D, Atala A, Sokern S (2013). Substrate elasticity controls cell proliferation, surface marker expression and motile phenotype in amniotic fluid-derived stem cells. J. Mech. Behav. Biomed. Mater..

[52] Yeung T, Georges PC, Flanagan LA (2005). Effects of substrate stiffness on cell morphology, cytoskeletal structure, and adhesion. Cell Motil. Cytoskelet..

[53] Dziob D, Kołodziej T, Nowak J (2016). Effect of substrate elasticity on macroscopic parameters of fish keratocyte migration. Phys. Biol..

[54] Trichet L, le Digabel J, Hawkins RJ (2012). Evidence of a large-scale mechanosensing mechanism for cellular adaptation to substrate stiffness. PNAS.

[55] Fritsch A, Höckel M, Kiessling T (2010). Are biomechanical changes necessary for tumour progression?. Nat. Phys..

[56] Qian M, Wang DC, Chen H, Cheng Y (2017). Detection of single cell heterogeneity in cancer. Semin. Cell Dev. Biol..

[57] Alexandrova, A.Y., Chikina, A.S., & Svitkina, T.M. Actin cytoskeleton in mesenchymal-to-amoeboid transition of cancer cells. in *International Review of Cell and Molecular Biology*. 197–256 (Elsevier Inc., 2020). 10.1016/bs.ircmb.2020.06.002 PMC9038018 33066874

[58] Graziani V, Rodriguez-Hernandez I, Maiques O, Sanz-Moreno V (2022). The amoeboid state as part of the epithelial-to-mesenchymal transition programme. Trends Cell Biol..

[59] Vaškovičová K, Szabadosová E, Čermák V (2015). PKCα promotes the mesenchymal to amoeboid transition and increases cancer cell invasiveness. BMC Cancer.

[60] Wolf K, Mazo I, Leung H (2003). Compensation mechanism in tumor cell migration: Mesenchymal–amoeboid transition after blocking of pericellular proteolysis. J. Cell Biol..

[61] Talkenberger K, Cavalcanti-Adam EA, Voss-Böhme A, Deutsch A (2017). Amoeboid–mesenchymal migration plasticity promotes invasion only in complex heterogeneous microenvironments. Sci. Rep..

[62] Hecht I, Bar-El Y, Balmer F (2015). Tumor invasion optimization by mesenchymal–amoeboid heterogeneity. Sci. Rep..

[63] Čermák V, Gandalovičová A, Merta L (2020). High-throughput transcriptomic and proteomic profiling of mesenchymal–amoeboid transition in 3D collagen. Sci. Data.

[64] Reimer M, Petrova Zustiak S, Sheth S, Martin Schober J (2018). Intrinsic response towards physiologic stiffness is cell-type dependent. Cell Biochem. Biophys..

[65] Ladoux B, Mège RM, Trepat X (2016). Front-rear polarization by mechanical cues: From single cells to tissues. Trends Cell Biol..

[66] Berg WA, Cosgrove DO, Caroline Doré MJ (2022). Shear-wave elastography improves the specificity of breast US: The BE1 multinational study of 939 masses 1 materials and methods. Radiology.

[67] Athanasiou A, Tardivon A, Tanter M (2010). Breast lesions: Quantitative elastography with supersonic shear imaging—Preliminary results. Radiology.

[68] Serra-Picamal X, Conte V, Vincent R (2012). Mechanical waves during tissue expansion. Nat. Phys..

[69] Wang, Y.-L., & Pelham, R.J. *Preparation of a Flexible, Porous Polyacrylamide Substrate for Mechanical Studies of Cultured Cells*. 489–496 (1998). 10.1016/s0076-6879(98)98041-7 9751904

[70] Beningo, K.A., Lo, C.-M., & Wang, Y.-L. *Flexible Polyacrylamide Substrata for the Analysis of Mechanical Interactions at Cell-Substratum Adhesions*. 325–339 (2002). 10.1016/s0091-679x(02)69021-1 12071003

[71] Seroussi I, Veikherman D, Ofer N (2012). Segmentation and tracking of live cells in phase-contrast images using directional gradient vector flow for snakes. J. Microsc..

[72] Entenberg, D., & Condeelis, J. *ROI Tracker. The ROI_Tracker Software was Supplied by David Entenberg and John Condeelis as Supported by CA100324 and GM064346.*

[73] Bear JE, Haugh JM (2014). Directed migration of mesenchymal cells: Where signaling and the cytoskeleton meet. Curr. Opin. Cell Biol..

[74] Lauffenburger, D.A., & Horwitz, A.F. *Cell Migration: Review A Physically Integrated Molecular Process* (1996). 10.1016/s0092-8674(00)81280-5 8608589

