# Supplementary material for: Impact of elastic substrate on the dynamic heterogeneity of WC256 Walker carcinosarcoma cells
Source: Sci Rep. 2023 Sep 21;13:15743. doi: 10.1038/s41598-023-35313-2 (PMC10514059; doi:10.1038/s41598-023-35313-2)
Supplement: Supplementary file 1 — Supplementary Legends. [file 41598_2023_35313_MOESM1_ESM.docx]

**Supplementary Information File for**

“Impact of Substrate Elasticity on the Dynamic Heterogeneity of WC256 Walker Carcinosarcoma Cells” by Aleksandra Mielnicka, Tomasz Kołodziej, Daniel Dziob, Sławomir Lasota, Jolanta Sroka, Zenon Rajfur.

**Legends:**

Supplementary Video S1. Subpopulations of investigated WC256 subline observed on (rigid) glass substrate. Scale bar: 50 µm, time stamp: minutes : seconds

Supplementary Video S2. Distinct types of cells included in mesenchymal subpopulation of investigated WC256 subline observed on (rigid) glass substrate. Scale bar: 50 µm, time stamp: minutes : seconds

Supplementary Video S3. Main subpopulational transitions observed for WC256 cells:

1. between mesenchymal and polygonal or bigonal subpopulations,
2. between polygonal or bigonal and amoeboid subpopulations.

Scale bar: 50 µm, time stamp: minutes : seconds
